# Editorial: Dysfunctional microglia in neurological disorders

**DOI:** 10.3389/fncel.2023.1133019

**Published:** 2023-01-24

**Authors:** Anup Bhusal, Arturo Ortega, Kyoungho Suk

**Affiliations:** ^1^Department of Pharmacology, School of Medicine, Kyungpook National University, Daegu, Republic of Korea; ^2^Department of Biomedical Science, School of Medicine, Kyungpook National University, Daegu, Republic of Korea; ^3^Department of Toxicology, Centro de Investigación y de Estudios Avanzados del Instituto Politécnico Nacional, Mexico City, Mexico; ^4^Brain Science and Engineering Institute, Kyungpook National University, Daegu, Republic of Korea

**Keywords:** microglial dysfunction, neurological disorders, reactivity states, microglial biology, neuroinflammation

Microglia are innate immune cells of the central nervous system that play crucial roles in maintaining brain health (Colonna and Butovsky, 2017). When exposed to pathogenic stimuli, these cells undergo changes in morphology, proliferation, and migration, which can contribute to neurodegenerative processes *via* the release of inflammatory mediators. It has been recently discovered that some microglial populations can impact the course or severity of neurological conditions, not only *via* inflammation, but also through a loss of normal microglial function, which hinders the ability to perform usual tasks, particularly in conditions such as Alzheimer's and Parkinson's diseases, mental disorders, and the normal aging process (Mosher and Wyss-Coray, 2014; Kam et al., 2020; Ayyubova, 2022; Bianchin and Snow, 2022). The specific cause of microglial dysfunction is not yet fully understood, but it has been suggested that intrinsic mutations in regulatory genes or metabolic abnormalities within microglia may be responsible (Aldana, 2019; Marschallinger et al., 2020; Wei and Li, 2022). However, this view of microglial dysfunction is limited to certain disease conditions, and a universal explanation for this hypothesis on dysfunctional microglia is lacking. This Research Topic aims to provide an overview of the current knowledge of microglial functions and dysfunctions as well as their distinct reactivity states in various pathological conditions, such as traumatic, ischemic, and hemorrhagic brain injury, multiple sclerosis, and neurocognitive disorders. This topic includes two research articles and four review articles covering various aspects of microglial biology and related pathophysiology.

Delbridge et al. demonstrated that brain slice cultures can provide a more physiologically relevant model for studying microglia than *in vitro* cultures of dissociated brain tissue. The use of RNA sequencing technology to examine microglial heterogeneity in various disease conditions has grown rapidly. RNA sequencing-based gene expression analysis revealed significant differences between the transcriptional signatures of microglia derived from *in vitro* cultures and acutely isolated adult microglia. Cultured microglia showed elevated expression of many genes that are rarely expressed or not expressed at all in freshly isolated adult microglia; these cultures also revealed low expression of microglia identity-related genes. In contrast, microglia from organotypic brain slice cultures showed greater similarity to adult microglia, with a higher expression of microglia-specific genes and less inflammation. Overall, this article found that organotypic brain slice culture preserves tissue architecture and cellular composition and provides a more physiologically relevant model for studying microglia. Although these findings are relevant, further research is needed to fully understand the advantages and limitations of this model in the context of brain injury and disease.

Another approach to studying microglial states is the use of reliable markers (Jurga et al., 2020). A study by Mercurio et al. showed that Tmem119, considered a protein specifically expressed by microglia, is downregulated in activated microglia after traumatic brain injury in mice. This decrease in Tmem119 immunoreactivity was observed in both amoeboid microglia, which are typically observed in the acute phase of traumatic brain injury, and hypertrophic microglia, which are found in the contused cortex. Surprisingly, while the protein levels of Tmem119 decreased, the expression of *Tmem119* mRNA was upregulated. This difference may have occurred because the *Tmem119* mRNA was not translated into proteins after traumatic brain injury, or because reactive microglia break down proteins faster after brain injury. Overall, this study highlights the need for novel markers that can accurately identify microglia in the context of brain injury.

This Research Topic also includes several review articles in which the authors express their opinions on different microglial states in various disease conditions. Yu et al. provided an update on the role of microglia in ischemic brain injuries. Microglial polarization is regulated by several signaling pathways, including NF-κB, Notch, mTORC1, AMPK, STAT, and PPAR-γ, which play a crucial role in determining the extent of damage caused by ischemic brain injuries. In addition, several drugs, including metformin, resveratrol, and anise alcohol, promote the transition between pro- and anti-inflammatory states. In some cases, microglial depletion using liposomal clodronate or Mac-1-saporin attenuates inflammation after ischemic brain injury. While further research is needed to fully understand these mechanisms and to determine the best manner to target microglia, microglia-specific modulation could display a promising outcome in the treatment of ischemic brain injuries.

Perioperative neurocognitive disorders are a group of conditions that affect cognitive function, including memory, attention, and language, in the period surrounding surgical procedures (Kong et al., 2022). Growing evidence suggests that microglia may be critically involved in the development of perioperative neurocognitive disorders. A plausible mechanism by which microglia may contribute to this condition is through the reactive state in response to systemic inflammation caused by surgical trauma. The use of general anesthetics such as sevoflurane may also transform microglia and contribute to this condition. Additionally, astrocyte-microglial communication and neuron-glial interactions may be involved. Overall, the review by Fan et al. suggests that microglia may be a key player in the development of perioperative neurocognitive disorders, and that further research is needed to understand the precise mechanisms underlying microglial involvement in this condition.

The dynamic role of microglia in animal models of multiple sclerosis was reviewed by Plastini et al.. Microglia exhibit a range of responses depending on the stage of multiple sclerosis. In the early stages of the disease, microglia become reactive and release inflammatory molecules that contribute to the destruction of the protective myelin coating around nerve fibers. However, during the chronic phase of the disease, microglia were shown to have protective functions that facilitate the repair process. This review highlights animal models that represent various aspects of multiple sclerosis and discusses the difficulty of accurately depicting the complexity and diversity of microglial responses in multiple sclerosis.

The last review of this Research Topic by Gu et al. draws attention to microglial pyroptosis. Pyroptosis is a form of programmed cell death triggered by the activation of caspases, which leads to the degradation of the cell's structural components and the release of pro-inflammatory molecules (Yu et al., 2021). The authors described the molecular processes involved in microglial pyroptosis and discussed the latest evidence on its role in intracerebral hemorrhage. They also highlighted potential treatments for intracerebral hemorrhage that target microglial pyroptosis.

Recent technological advances have led to significant progress in the study of microglia. It has become evident that the complex states of microglia are not sufficiently captured by the concept of pro- and anti-inflammatory states. Latest studies have identified multiple microglial states, which include lipid droplet-accumulating microglia, disease-associated microglia, neurodegenerative microglia, dark microglia, proliferative-region-associated microglia, white matter-associated microglia, activated response microglia, necroptotic microglia, late-response microglia, and dystrophic microglia in a variety of neurodegenerative conditions (Wei and Li, 2022). Various microglial states have been identified based on their distinct morphologies or transcriptional profiles under different neurological conditions. It has been suggested that there is another state of microglia, in which reduced function—rather than reactivity or gain of function—contributes to disease development (Bianchin and Snow, 2022; Neumann et al., 2023). These microglia are thought to be exhausted, overstimulated, or overused during inflammatory reactions, and may not function effectively. However, it is not clear how to categorize these microglia, and there is no clear consensus about whether the term “dysfunctional microglia” refers to a specific subtype or is a general term that includes all of the aforementioned microglia. Therefore, it is crucial to define microglial subpopulations and identify their precise functions to better understand various neurological diseases. We also must be cautious when assigning names to different types of microglia based on studies using technologies such as microarrays and next-generation sequencing, as these technologies are frequently updated and the names used to describe microglia may quickly become outdated. Furthermore, when using omics approaches, particularly those involving RNA sequencing, there are several limitations to consider. One of the most significant limitations is that levels of RNA and proteins are not directly equivalent. While RNA serves as a template for protein synthesis, the final protein product can be altered post-translationally, generating diverse proteoforms and altering its function. Thus, changes in RNA levels do not always correlate with changes in protein levels or activity as shown by Mercurio et al.. Another limitation is that these approaches are typically performed *ex vivo*. This can introduce artifacts and make it difficult to accurately reflect the *in vivo* state as suggested by Delbridge et al.. In comparison, *in situ* or *in vivo* approaches can provide more accurate and relevant information, as they more closely mimic the natural environment and conditions in which the biological processes occur. On the other hand, the use of recently developed microglia-specific markers may not be reliable for identifying these microglia, as shown by Mercurio et al., and it may be necessary to develop novel markers that accurately define distinct microglial states. Overall, the articles included in this Research Topic provide a comprehensive overview of the current knowledge of microglial function and dysfunction, and will be of interest to both glial researchers and the wider community of neuroscientists.

## Author contributions

AB and KS wrote the manuscript. AO revised and edited the final version. All authors contributed to the article and approved the submitted version.
